# Alzheimer's Association Project VITAL: A Florida Statewide Initiative Using Technology to Impact Social Isolation and Well-Being

**DOI:** 10.3389/fpubh.2021.720180

**Published:** 2021-12-02

**Authors:** Lorna E. Prophater, Sam Fazio, Lydia T. Nguyen, Gizem Hueluer, Lindsay J. Peterson, Kasia Sherwin, Julie Shatzer, Michelle Branham, Amy Kavalec, Karen O'Hern, Kristi Stoglin, Rio Tate, Kathryn Hyer

**Affiliations:** ^1^Alzheimer's Association, Chicago, IL, United States; ^2^iN2L, Greenwood Village, CO, United States; ^3^School of Aging Studies, University of South Florida, Tampa, FL, United States

**Keywords:** Alzheimer's, dementia, technology, social isolation, COVID-19, tablets

## Abstract

Lack of social engagement and the resulting social isolation can have negative impacts on health and well-being, especially in senior care communities and for those living with dementia. Project VITAL leverages technology and community resources to create a network for connection, engagement, education, and support of individuals with dementia and their caregivers, and explores the impact of these interventions in reducing feelings of social isolation and increasing mood among residents during the COVID-19 pandemic. Through two phases, 600 personalized Wi-Fi-enabled iN2L tablets were distributed to 300 senior care communities (55% assisted living communities, 37% skilled nursing communities, 6% memory care communities, and 2% adult family-care homes) to connect and engage residents and their families. Different phases also included Project ECHO, a video-based learning platform, Alzheimer's Association virtual and online education and support for family caregivers, evidence-based online professional dementia care staff training and certification, and Virtual Forums designed to explore ways to build sustainable, scalable models to ensure access to support and decrease social isolation in the future. Tablet usage was collected over an 11-month period and an interim survey was designed to assess the effectiveness of the tablets, in preventing social isolation and increasing mood among residents during the COVID-19 pandemic. A total of 105 care community staff (whose community used the tablets) completed the survey and overall, these staff showed a high level of agreement to statements indicating that residents struggled with loneliness and mood, and that the tablet was useful in improving loneliness and mood in residents and allowing them to stay in touch with family and friends. Additional positive results were seen through a variety of other responses around the tablets and Project ECHO. Overall, the tablets were shown to be an effective way to engage residents and connect them with friends and family, as well as being a useful tool for staff members. A third phase is currently underway in the homes of people with dementia and their family caregivers, which includes tablets and direct access to Alzheimer's Association virtual and online education and support programs.

## Introduction

Social engagement is an important aspect of well-being and cognitive health, especially in older adults (1–3). Conversely, the lack of social engagement and the resulting social isolation can have negative impacts on health and well-being (4–6). Nicholson (5) reviewed 74 publications and concluded that social isolation is an under assessed condition in older adults and has a number of detrimental outcomes including those that are physical, psychological, and physiological. Currently, it is estimated that 17% of adults aged 65 and older are socially isolated resulting in a 26% increased risk of early death due to subjective feelings of loneliness (7).

Loneliness and social isolation are an even greater concern for those in senior living environments. Severe loneliness is at least twice the rate for those in senior living communities than in the general community (8). Cudjoe et al. (9) found that isolation can impact both emotional and physical well-being of residents based on the decrease of social connections through events including geographic migration of children, relatives, or friends; death or disability among social network members; and personal factors including decline in physical or cognitive abilities.

In the recent face of COVID-19, the impact of social isolation in senior living communities has only increased with the social distancing guidelines, cancelation of group and communal activities, and closure to visitors, all resulting in a significant increase in isolation and the resulting loneliness felt by residents (8). The restrictions implemented during COVID-19 have disrupted the ability for residents to connect with their usual support systems and increased distressing behaviors and mood disturbance especially for those with Alzheimer's disease and related dementia (ADRD) (10). Additionally, Ray (10) suggests that during a crisis such as COVID-19, care community leaders and clinicians should be cognizant of the psychological well-being of residents as much as their chronic medical conditions.

To mitigate these risks and to support those in senior living communities, especially those living with ADRD, the potential utility of technology has increasingly been explored. A number of technologies have shown a positive impact on reducing social isolation, increasing quality of life, increasing positive emotions, and promoting greater level of activity and social engagement (11, 12). The purpose of this project is to assess the effectiveness of technology, specifically tablets, in reducing feelings of social isolation and increasing mood among residents during the COVID-19 pandemic.

## Context

Project VITAL (Virtually Inclusive Technology for ALl) is a unique project that leverages technology and community resources to create a network for connection, engagement, education, and support of individuals with ADRD and their caregivers. This combination of components aims to positively impact social isolation, stress, and well-being. Originally launched in April 2020 with additional phases continuing into 2021, Project VITAL is a public-private partnership between Florida's Department of Elder Affairs (DoEA), the Alzheimer's Association, and other stakeholders, to help mitigate the effects of isolation during the COVID-19 pandemic and beyond.

Phase One (VITAL 1.0) included three components to impact connection, engagement, education, and support. The first included the distribution of 300 personalized tablets in 150 care communities to connect and engage residents and their families. The second component was Project ECHO (Extension for Community Healthcare Outcomes), implemented to facilitate educational and support opportunities for staff through a video-based learning platform. The third component was Alzheimer's Association virtual and online education and support for family caregivers in targeted underserved communities.

Phase Two (VITAL 2.0) rolled out in June of 2020 with an expansion to an additional 150 care communities and 300 additional tablets. VITAL 2.0 added two additional components. The first component was evidence-based online professional dementia care staff training and certification. The second component was VITAL Virtual Forums, designed to engage stakeholders in exploring ways to build sustainable, scalable models for increasing access to support and decreasing social isolation in the future for all Floridians.

VITAL 1.0 and VITAL 2.0 combined, involved a total of 300 care communities comprised of assisted living communities (55%), skilled nursing communities (37%), memory care communities (6%), and adult family-care homes (2%). Of these communities, 14% had 1–9 beds, 15% had 10–49 beds, 31% had 50–99 beds, 29% had 100–149 beds, and 11% had more than 150 beds. Each community had a lead contact and a back-up point of contact for the project who also completed the evaluation survey.

## Key Programmatic Elements

### iN2L Tablets

Tablets were a core component within the VITAL projects. The technology secured from iN2L were Samsung Galaxy tablets, pre-programmed with iN2L proprietary software aimed to facilitate connections between residents living with ADRD and families through various means. The tablet device was Wi-Fi-enabled and was created to be intuitive and simple to use and included security features to keep seniors safe during use. Residents and staff had no need for any previous smartphone or tablet experience. Staff received initial training and on-going support to implement the individualized programming and engagement with families. The interface provided simple touch access to an array of content specifically designed and curated for older adults, such as games, puzzles, trivia, music, sing-alongs, music therapy, audiobooks, movies and TV shows, virtual tours, history, and spiritual content. The tablets were also equipped with applications for direct video call connection to residents' family members, COVID-19 information and tips, and Alzheimer's Association programs, services, and resources. The tablets allowed content to be tailored to the residents' likes and interests and provided single touch connectivity for video calls.

### Project ECHO

Project ECHO® was included as part of VITAL 1.0 and 2.0 and is an evidence-based distance-learning model that builds workforce capacity to provide best practice care (historically in rural and underserved communities). In this model, a team of multidisciplinary experts come together with community-based partners in regularly scheduled collaborative learning sessions to participate in case-based discussions and hear experts present on best-practice care. Long-term care providers gain knowledge, confidence, and access to specialist consultation so that they can deliver excellent care to residents in their own care communities. Information flows in multiple directions: community care teams learn from specialists, specialists learn from community care teams on the front lines, and everyone learns from their peers.

Three separate learning cohorts completed five sessions each, and discussed examples from their care communities and had the opportunity to hear from other care communities facing similar challenges. Thirty-two assisted living communities signed up for the series and 15 completed the program.

### Professional Training and Certification

The Alzheimer's Association Person-Centered Dementia Care Training Program educates professional care workers in long-term and community-based care settings on current evidence-based, person-centered practices to care for individuals living with dementia.

It is a self-paced, online training that provides 4 h of educational content and covers foundational information on Alzheimer's and dementia and four topic areas of the Dementia Care Practice Recommendations, which serve as the benchmark for quality care across the disease spectrum:

- Alzheimer's disease and dementia
- Person-centered care
- Assessment and care planning
- Activities of daily living
- Dementia-related behaviors and communication

After completing the online training program, staff have access to the Alzheimer's Association's essentiALZ®, an individual exam that demonstrates knowledge of quality care dementia practices. Staff who pass the exam are certified in essentiALZ for 2 years.

### VITAL Virtual Forums

VITAL Virtual Forums were created to explore the complex reality of accessing care and decreasing social isolation specifically in Florida with invested and interested stakeholders. Attendees came together through Zoom to engage in virtual discussions and presentations and included caregivers, industry administrators, professional care providers, state aging units, and the general public. Three Project VITAL Virtual Forums (each 2 h) were held between August 2020 and February 2021 with an additional forum planned for August 2021.

## Evaluation

### iN2L Tablet Usage

iN2L tablet usage data was collected as part of Project VITAL. The tablet usage data discussed in this article was collected over an 11-month period from May 2020 through March 2021. The May and June usage data included VITAL 1.0 data (150 communities; 300 tablets total), and July through March usage data included both VITAL 1.0 and VITAL 2.0 data (300 communities; 600 tablets total). The VITAL 1.0 and VITAL 2.0 data reported in this section use averages across the full 11-month period (May 2020–March 2021).

#### Overall Usage

Staff members provided residents with access to the tablets. Each resident could access their own profile with a simple passcode. This allowed the resident to initiate one-touch video calls, access their personal phonebook, view photos sent from family and friends, and access their favorite content. User sessions are defined as a distinct use of the tablet between a profile login and logout period. On average, a single user session included approximately three different activities (e.g., a video call followed by a playing game and then listening to music). There were an average of 4,140 user sessions per month with an average session time of 34 min. The average session time was calculated using a weighted average to account for differences in the total number of sessions each month. The weighted average was computed as follows:


$$\begin{array}{l} \frac{\sum_{m=1}^{m}s_{m}\;*\;t_{m}}{\sum_{m=1}^{m}s_{m}} \end{array}$$

where *m* = number of months, *s*_*m*_ = number of sessions in the given month, and *t*_*m*_ = average session time in the given month.

#### Video Calls

There were an average of 1,995 video calls each month with an average video call time of 7 min. The average video call time was calculated using a weighted average, as described above.

#### Content

The tablet has an extensive content library specifically for older adults, with more than 1,000 items that promote wellness and engagement and that can accommodate different levels of cognitive ability. There were an average of 10,343 content sessions each month with an average content session time of 14 min. The average content time reflects a weighted average, calculated using a procedure like the one described above. On average, 926 unique content items were accessed each month. The top 5 most popular content items across the 11-month period were (1) Puzzles, (2) Solitaire, (3) Therapeutic music, (4) Word Grid game, and (5) Bubble Popper. The tablets also include easy home screen access to the Alzheimer's Association's online educational content. This material was accessed on average 48 times each month. From May to October 2020, the tablets provided information on COVID-19 and during that time, that content was accessed an average of 466 times per month.

### Interim Tablet Use Survey

The evaluation survey was designed to assess the effectiveness of technology, specifically the tablets, in preventing social isolation and increasing mood among residents during the COVID-19 pandemic. The survey was administered *via* Qualtrics to VITAL 1.0 and 2.0 care community staff members and included questions related to demographics, employment, implementation of the tablet, and staff perceptions regarding effectiveness, training and support, ease of use, and acceptance. In care communities with a separate unit for ADRD (61 communities), staff perceptions regarding effectiveness were assessed separately for residents without, vs. with, ADRD. Staff members at participating care communities were invited to respond to the survey in two phases (VITAL 1.0 and VITAL 2.0). Staff were invited to participate in the survey ~6 months after implementation. VITAL 1.0 staff received their invitation in November 2020 and VITAL 2.0 staff received their invitation in January 2021. Invitations were sent by email to community points of contact.

A total of 107 staff completed the survey. Two staff (one from a VITAL 1.0 and one from a VITAL 2.0 care community) indicated that their communities did not use the tablets and were excluded from analysis. In total, complete survey responses were available from 62 staff from VITAL 1.0 communities and 43 from VITAL 2.0 communities for a total of 105 staff responses. Responses were pooled across VITAL 1.0 and 2.0 care communities because an initial analysis indicated few differences by phase. The average age for staff in the survey was 49 years (SD = 12 years), and 91 staff identified as women, 12 identified as men, and 2 did not indicate their gender. Of all staff, 68 identified as White, 10 identified as Black or African American, 10 identified as Hispanic or Latino, 3 identified as Asian, 7 identified as Other, and 7 opted not to identify. On average, staff worked in their respective care community for 6 years (SD = 7 years). Sixty-four were activity directors, 19 were administrators, 11 held other positions, and 3 opted not to answer. Eleven staff had a master's degree, 44 completed a 4-year college degree, 15 completed a 2-year college degree, 22 completed high school, and 8 opted not to answer.

Table 1 gives an overview of staff members' perceptions regarding residents' adjustment to COVID-19 precautionary measures and effectiveness of the tablets. Sixty-one care communities had a special unit for residents with ADRD, while 44 did not. Data are provided separately by whether the care community had a unit for residents with ADRD, and if so, whether the responses applied to residents without or with ADRD. Overall, staff members showed a high level of agreement to statements indicating that residents struggled with loneliness and mood since the beginning of the COVID-19 pandemic. Staff also showed high levels of agreement to statements indicating that the tablet was useful in improving loneliness and mood in residents and allowing them to stay in touch with family and friends. Staff showed moderate levels of agreement to statements that the tablet improved residents' adjustment to COVID-19 precautionary measures, that residents understood how to use the tablets, and that staff had enough time to help the residents with the tablets. A series of paired-sample *t*-tests were performed to examine whether perceptions differed for residents without vs. with ADRD in communities that included a unit for ADRD. A significant difference was found (at *p* < 0.05, two-sided) for the statement concerning residents' understanding of tablet use, indicating lower levels of agreement that residents with ADRD understood how to use the tablets, *t*_(60)_ = −4.40, *p* < 0.01. This finding is not unexpected as the tablets are designed for staff to use the tablet alongside residents with ADRD. There were no significant differences in the agreement to other statements.

**Table 1:** Staff perceptions on mood, loneliness, and tablet use (on a scale from 1 “strongly disagree” to 5 “strongly agree”).

**Statement**	**Communities without an ADRD unit**		**Communities with an ADRD unit—residents** ***without*** **ADRD**		**Communities with an ADRD unit—residents** ***with*** **ADRD**	
	**Mean**	**SD**	**Mean**	**SD**	**Mean**	**SD**
Residents have been struggling with loneliness since the implementation of the COVID-19 precautionary measures	3.98	1.32	4.25	0.96	4.38	0.84
Residents' mood declined since the implementation of the COVID-19 precautionary measures	3.86	1.27	4.33	0.83	4.20	0.95
The iN2L tablets were useful in reducing residents' loneliness	3.93	1.15	4.13	1.04	4.28	0.92
The iN2L tablets were useful in improving residents' mood	4.09	1.01	4.20	0.95	4.31	0.90
The iN2L tablets made it easier for residents to stay in touch with family and friends	4.00	1.22	4.18	0.97	4.30	0.99
Residents enjoyed using the iN2L tablets	4.16	1.03	4.18	1.01	4.30	0.95
The iN2L tablets made it easier for residents to adjust to COVID-19 precautionary measures	3.61	1.02	3.93	1.01	3.87	1.06
Residents understood how to use the iN2L tablets	3.34	0.99	3.36	1.16	2.92	0.94
I had enough time to help residents with the iN2L tablets	3.55	1.27	3.70	1.02	3.64	1.14

*ADRD, Alzheimer's disease and related dementias*.

Table 2 gives an overview of responses regarding leadership, training and support, ease of use, acceptance, and recommendation. The leadership was stable in most care communities and was perceived as highly supportive of the tablets. Staff also indicated high levels of agreement that they received sufficient training and technical support for the tablets, and that the tablets were easy to implement in daily routines and were easy to use. Staff showed very high levels of agreement to the statements that they enjoyed using the tablets with the residents and that they welcomed them. On average, staff indicated that it was very likely they would recommend the tablets to friends and colleagues.

**Table 2:** Staff perceptions on leadership, training and support, ease of use, acceptance, and recommendation.

**Statement**	**Agreement**	
	**Mean or %**	***SD***
**Leadership (from 1 “strongly disagree” to 5 “strongly agree”)**		
Leadership changed in the last 6 months	17%	
Executive leadership supported the iN2L tablets	4.50	0.95
**Training and support (from 1 “strongly disagree” to 5 “strongly agree”)**		
I had sufficient training on the use of the iN2L tablets	4.14	1.13
I had sufficient technical support on the use of the iN2L tablets	4.18	1.12
**Ease of use (from 1 “strongly disagree” to 5 “strongly agree”)**		
The iN2L tablets were easy to include in our daily routines	4.00	1.14
The iN2L tablets were easy to use	4.23	1.05
**Acceptance (from 1 “strongly disagree” to 5 “strongly agree”)**		
I enjoy using the iN2L tablets with the residents	4.46	0.93
I welcome the use of the iN2L tablets	4.58	0.87
**Recommendation (from 0 “very unlikely” to 10 “very likely”)**		
How likely are you to recommend the iN2L program to friends and colleagues?	8.60	1.86

Taken together, the results of this survey indicate that the tablets were perceived favorably by staff in VITAL 1.0 and 2.0 communities. Importantly, staff perceptions were equally favorable both for residents without and with ADRD. These findings suggest that the tablet may be a valuable tool to reduce social isolation and to improve mood in senior living communities.

### Project ECHO

A post-survey was conducted with staff members who participated in Project ECHO. A total of 13 staff members completed the evaluation across the three cohorts from the 15 communities who participated and completed ECHO. It is not possible to determine if the 13 staff members were from 13 unique communities or not, which is a limitation of the design and should be addressed in future iterations.

All 13 staff members who completed the Project Echo evaluation were satisfied with the program, and found the information learned in Project ECHO was valuable in their work. In addition, 62% of participants made changes in the way they care for community members based on what they learned, and 78% said Project ECHO improved the quality of care they provide to community members with dementia. Lastly, 78% of participants said Project ECHO positively influenced the way they interacted with families and caregivers of community members with dementia.

### Professional Training and Certification

Launched in January 2021 as a supplement to Project VITAL, the training and certification was met with interest from direct care workers in settings such as assisted living, home care, skilled nursing, adult day, and hospice. As of April 30th, 452 people had completed an interest form for the Project VITAL essentiALZ training and certification. Deployed in cohorts, the March 1st cohort had 150 participants, the April 1st cohort had 100 participants, and the May 1st cohort had 75 participants. The goal is to train and certify a total of 1,200 professional care providers.

To date, 141 individuals of the 325 in deployed cohorts have completed the training and certification and have submitted their evaluation. The other 184 individuals had not yet completed the training and certification, and thus did not have access to the evaluation at the time data were analyzed. Of the 141 individuals who completed the evaluation, 125 responded they were very satisfied with the training, 9 were somewhat satisfied, 4 were very dissatisfied and 3 opted to not respond. In addition, 112 of the 141 strongly agreed the training gave them important information about how to give person-centered care, 17 somewhat agreed, 3 neither agreed nor disagreed, 2 strongly disagreed, and 7 opted not to respond.

### VITAL Virtual Forums

The VITAL Virtual Forums addressed the overview and progress of VITAL and included “Combating Social Isolation for at Home and Long Term Facility Care” with 389 attendees, “Florida Advocacy - A Vital Update” co-hosted by the Department of Elder Affairs with 117 attendees, and “The Road Ahead: Project Vital Update and Advocacy Events” with 160 attendees. No formal evaluation was conducted.

## Discussion

To effect positive change in the lives of individuals with dementia and the staff that provide care, Project VITAL 1.0 and 2.0 implemented customized, senior-friendly tablets in senior care communities, along with Project ECHO, virtual and online education support, and VITAL virtual forums.

The tablets were shown to be an effective way to engage and connect residents, as well as being a useful tool for staff members. The findings related to the tablets help demonstrate the value that purpose-built, senior-friendly technology can bring to older adults' lives, particularly in care communities. The tablet usage data demonstrates that residents used the tablets frequently and that they remained engaged with the tablet each time they used it. The usage seen here also speaks to the growing body of work showing that when older adults are given access to technology that has been designed specifically for them, they are interested, willing, and able to learn and use such technology (13–15).

Additionally, the tablets helped staff to proactively connect residents with their families without a significant impact on staff time, as well as address family's questions or concerns by allowing families to see residents through video calls. This technology-based connection and engagement can help provide families with ongoing confidence and comfort. The video call data suggests that older adult residents are interested in using this technology to connect with others, provided they are given access to the tablet. To better understand the specific value of the video call feature for older adults, future evaluations may benefit from obtaining insights into who residents spoke with on video calls (e.g., are they repeatedly connecting with existing contacts; is their social network growing?) and/or the purpose of the video calls (e.g., connection vs. telehealth).

The content items that were accessed most frequently suggest that the tablets are commonly used for cognitive engagement and relaxation. The regular use of the tablets for such purposes has important implications. Engagement in cognitive leisure activities by older adults, which include puzzles and games, has been related to better cognition (16–18), reduced risk of dementia or cognitive decline [reviews and meta-analysis (19, 20)], and better mental health (16). Additionally, the prominence of games among the most popular content items likely speaks to the importance of design considerations for promoting adoption and use of technology in this population.

The COVID-19 pandemic has highlighted the value of technology, particularly for senior living operators who have increasingly recognized that technology is a necessity moving forward and not simply an amenity. A 2020 report by iN2L found a 60% increase in the number of senior living operators who think that engagement technology is extremely important now vs. pre-pandemic, and a 100% increase in the number of operators who see a definitive return on investment for engagement technology (21). Collectively, these findings suggest that technology needs to be ubiquitous and accessible across care communities to benefit the well-being of residents, families, and staff.

In addition to technology, Project VITAL included additional components to support person-centered care practices, more specifically engagement and connection. Professional training and certification provided the foundation knowledge of quality care practices based on the evidence-based Alzheimer's Association Dementia Care Practice Recommendations. Additionally, Project ECHO provided case-based learning and support as care teams made sustainable changes within their communities. Lastly, Virtual Forums engaged stakeholders in exploring ways to build sustainable, scalable models for increasing access to support and decreasing social isolation in the future.

There are a few limitations to note for this study. First, for the care community surveys, there was a relatively low response rate with 105 staff from the 300 participating care communities who used the tablets, which may limit the generalizability of our findings. The low response rate may be due in part to the design of the project, in which completing evaluations was not a requirement for participation. Although the staff responses still provide valuable insights, it will be important for future projects to incorporate a survey requirement for project participation. For example, it is possible that staff who were positive in their assessments were more likely to complete the evaluation than those who did not have good experiences with the program. Additionally, many staff members of these care communities have been overwhelmed by additional requirements and adjustments that have been needed to deal with the COVID-19 pandemic. As such, part of our lower response rate could be due to staff members feeling as if they do not have time to complete the survey. Second, staff responded with their perceptions of the residents' mood, feelings of loneliness, and table usage. It is possible that responses to these questions depend on how well staff know the residents. Third, as part of the project design, the surveys were anonymous. The survey included a question on the staff member's role/job title and whether their care community has a memory care unit, but there was not a question about the type of community they work at (e.g., assisted living) or more specifics about their residents (number, demographics, etc.). Thus, we cannot examine potential differences that may exist between community types, such as those that may arise from differences in level of care. Future iterations should collect such information to allow for comparison across groups. Fourth, we were not able to collect data prior to the implementation of the tablets. This would have allowed us to directly compare some measures before and after the implementation (for example, those related to the mood of residents and social isolation). Lastly, the lack of demographics and evaluation for the VITAL Virtual Forums impeded the ability to capture learnings and benefits. In the future, including demographics in the sign-up (including role, type of work, and community type) and a post-evaluation would be beneficial.

Given the positive findings from VITAL 1.0 and 2.0, the next question is whether such a program implemented in the homes of people with ADRD and their family caregivers can meaningfully impact social isolation, well-being, and stress. Accordingly, a third phase of Project VITAL is underway (Project VITAL At Home) in the homes of people with ADRD and their family caregivers and includes iN2L tablets and direct access to Alzheimer's Association virtual and online education and support programs. With nearly 60% of individuals living with dementia being cared for in the home (22), the impact of programs like Project VITAL can be monumental.

## Data Availability Statement

The raw data supporting the conclusions of this article will be made available by the authors upon request, without undue reservation.

## Ethics Statement

Ethical approval for this study and written informed consent from the participants of the study were not required in accordance with local legislation and national guidelines. The University of South Florida IRB determined that the proposed activity did not constitute research involving human subjects as defined by DHHS and FDA regulations.

## Author Contributions

KH and SF contributed to the conception and design of the study. GH, LTN, RT, and LEP created the evaluation survey. GH and RT performed the statistical analysis. LEP and SF wrote the outline and contributed to sections of the manuscript. LTN, GH, LJP, and KSh wrote sections of the manuscript. MB, JS, AK, KSt, and KO provided insight to content for sections of the manuscript. All authors contributed to manuscript revisions, and approval of the submitted version.

## Funding

Funding for Project VITAL was provided by the Florida Department of Elder Affairs (Contract XQVID) to create a network for connection, engagement, education and support of individuals with dementia and their families/caregivers to positively impact social isolation, stress, and well-being.

## Conflict of Interest

The authors declare that the research was conducted in the absence of any commercial or financial relationships that could be construed as a potential conflict of interest.

## Publisher's Note

All claims expressed in this article are solely those of the authors and do not necessarily represent those of their affiliated organizations, or those of the publisher, the editors and the reviewers. Any product that may be evaluated in this article, or claim that may be made by its manufacturer, is not guaranteed or endorsed by the publisher.
